# Etymologia: *Klebsiella *

**DOI:** 10.3201/eid1609.ET1609

**Published:** 2010-09

## [kleb´´el´?]

The genus *Klebsiella*, family Enterobacteriaceae, was named by V. Trevisan in 1885 in honor of German bacteriologist Theodor Albrecht Edwin Klebs (1834–1913). Dr Klebs is known for his pioneering work demonstrating that microorganisms are responsible for infectious diseases. He also studied the pathologic and bacteriologic features of gunshot wounds; investigated tuberculosis and successfully transmitted the disease to cattle; did research on the bacteriologic characteristics of malaria and anthrax; and, with Friedrich A.J. Löffler in 1884, discovered the etiologic agent of diphtheria, first called the Klebs-Löffler bacillus (later called *Corynebacterium diphtheriae*).

Sources: Dworkin M, Falkow S, Rosenberg E, Schleifer K-H, Stackebrandt E. The prokaryotes. *Klebsiella*. New York: Springer; 2006. p. 159–96; Carter KC. Koch’s postulates in relation to the work of Jacob Henle and Edwin Klebs. Medical History. 1985;29:353–74; Dorland’s illustrated medical dictionary, 31st edition. Philadelphia: Saunders Elsevier; 2007.

